# Twenty Years of Targeted Treatment in Rheumatoid Arthritis in the Greek Databases: Achievements and Unmet Needs

**DOI:** 10.31138/mjr.30.3.141

**Published:** 2019-09-30

**Authors:** Prodromos Sidiropoulos, Petros P. Sfikakis, Dimitrios D. Boumpas, Dimitrios Vassilopoulos

**Affiliations:** 1 Department of Rheumatology and Clinical Immunology, School of Medicine, University of Crete, Greece,; 2 First Department of Propaedeutic and Internal Medicine & Rheumatology Unit, School of Medicine, National and Kapodistrian University of Athens, Athens, Greece,; 3 4th Department of Internal Medicine, School of Medicine, National & Kapodistrian University of Athens, Athens, Greece,; 4 2nd Department of Medicine and Laboratory, Hippokration General Hospital, Medical School, National and Kapodistrian University of Athens, School of Medicine, Athens, Greece,; 5 Joint Rheumatology Program, National and Kapodistrian University of Athens, School of Medicine, Athens, Greece,; 6 Laboratory of Rheumatology, Inflammation and Autoimmunity, Institute of Molecular Biology and Biotechnology, Foundation for Research and Technology, Heraklion, Greece,; 7 Laboratory of Immune Regulation and Tolerance, Autoimmunity and Inflammation, Biomedical Research Foundation of the Academy of Athens, Athens, Greece

**Keywords:** biologic agents, rheumatoid arthritis, spondylarthritis, registry

## Abstract

Rheumatoid arthritis (RA) is a chronic autoimmune disease associated with substantial morbidity and mortality especially in difficult to treat cases. Biologic agents were introduced 20 years ago in Greece and RA management has paralleled the European experience. Several publications from the country have captured important aspects of the disease from its epidemiology to the clinical use of biologics and management of comorbidities. In this communication we review the management of RA and its evolution over the last 20 years in Greece, discussing the major achievements and the unmet needs of the disease in an effort to put this into a perspective. We conclude that introduction of biologic therapy has substantially changed the treatment of difficult to treat rheumatoid arthritis in-spite of the multiple unmet needs. While striving for even better outcomes, we cannot lose sight of the major impact of biologic therapies on the lives of patients with rheumatoid arthritis.

## THE HELLENIC REGISTRY OF BIOLOGIC THERAPIES (HeRBT)

**Figure 1.**
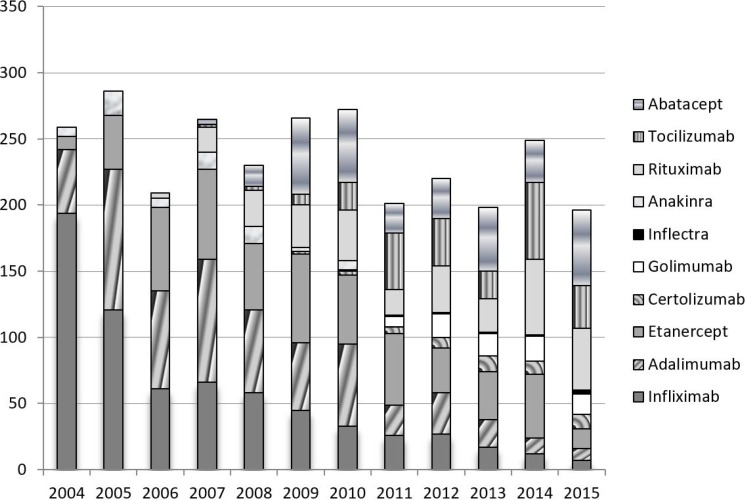
Trend of all bDMARDs treatments (either as a 1st or after bDMARD switches) recorded yearly in HeRBT.

**Figure 2.**
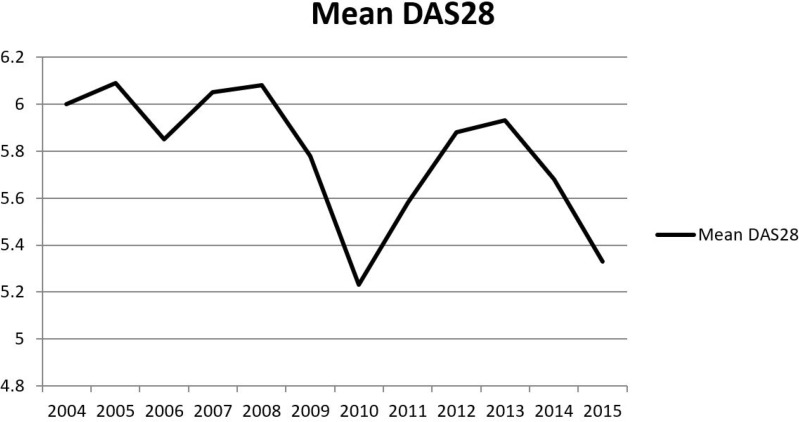
Mean DAS28 of RA patients at bDMARD initiation during the follow-up.

Early on after the introduction of biologic agents in Greece, the rheumatology community established the Hellenic Registry of Biologic Therapies (HeRBT), a nationwide, prospective, observational study that included patients with inflammatory arthritides starting bDMARDs, under the auspices of the Greek Rheumatology Society and Professional Association of Rheumatologists (ERE-EPERE). Reports from HeRBT capture current treatment practices in Greece and provide with novel knowledge rheumatology community about drug survival, toxicities and predictors of response.^5–9^ Eight academic and national health system rheumatology referral centers located in 5 cities of Greece (three in northern Greece, one in western, one in southern Greece and three in Athens) participated, targeting to represent both urban and rural population dispersed in different geographical areas of Greece. All consecutive patients followed in the participating centers who started their first biologic agent were invited to participate, thus minimizing the risk of selection bias. Enrolment in HeRBT was started in January 2004 and closed in May 2015. More than 95% of patients on bDMARDs followed in the participating centers were included. All patients were followed in hospital setting and no private practitioners were involved in the project; a fact that limits the external validity of the study. As this was an observational study, patients had unrestricted access to bDMARD agents based on the decision made by their treating physician and in accordance with the Hellenic Society of Rheumatology recommendations. Paper case report forms (CRFs) with demographic, clinical, laboratory and patient-reported variables were completed during routine patient evaluation at fixed time-points, according to the protocol. Any withdrawal from bDMARD treatment was registered prospectively, and treating physician was reporting the cause of withdrawal among a predefined list of causes. Additionally, treating physicians were urged to record and describe all events during the follow-up, which was then categorized in the database according to the Rheumatology Common Toxicity Criteria. By the end of 2015, 2874 patients had been included in the Registry (total follow-up time: 14445 patient-years), 1608 of whom had RA. The biologic treatment courses registered were 4352 in total (2855 for RA). Biologic agents used through the years in HeRBT are depicted in *Figure 1*, while mean DAS28 of RA patients starting a bDMARD is shown in *Figure 2*.

In RA, an analysis from HeRBT for 1.028 patients treated with anti-TNFα agents (median follow up of 3 years for infliximab, etanercept and adalimumab) showed that more that 40% of patients with established RA had erosive disease, the median DAS28 was in the level of high disease activity and median HAQ was 1 at the time of starting an anti-TNFα biologic.^7^ At twelve months, the rates of DAS28 remission ranged between 15–23% and of low disease activity between 27–34%, with overall drug survival rates ranging between 64–68% at 1 year and 31%–49% at 5 years. As expected, patients started on second and third TNF-α inhibitor had lower efficacy-related survival compared to anti-TNFα naïve patients. Both the levels of baseline disease activity and the achievement of treatment targets were within the range of cohorts from various countries, reflecting comparable burden of disease and therapeutic results. Importantly, data from the same cohort showed that 90% of the patients with improvement in inflammatory burden after TNFis showed significant improvement in quality of life (based on Euroqol questionnaire), a domain of interest for patients and payers.^5^

## DATA FROM THE E-PRESCRIPTION AND THE TREATMENT AND CO-MORBIDITIES REGISTRY OF THE HELLENIC SOCIETY OF RHEUMATOLOGY (ERE-EPERE)

After the wide-spread use of TNFα inhibitors in clinical practice, successive approval of non-TNFα inhibitors marked another major change in the guidelines and everyday management of RA, offering additional treatment options for the cases with inadequate response or toxicities to TNFis. Reports from Greek centers have reported on non-TNFi effectiveness, safety and predictors in clinical practice.^10–16^ Following the financial crisis in Greece, a nation-wide e-prescription platform was introduced, which captures the prescribing patterns at nationwide level. A co-operation of the academic centers with the healthcare authorities resulted in the analysis of the data from this administrative database of bDMARDs prescriptions, that captured almost all patients with RA under biologics. This analysis of 2017 with 9824 patients, showed that 73,4% of these patients were taking TNFα inhibitors, 18,71% of all patients were on biologic mono-therapy and the switching rate during one year of follow up was 7,73%.^17^ The estimate for biologic penetration was 19%, indicating a modest biologic use.

The more recent active country-wide database is the one created by the RA working group (RA-WG) of the Hellenic Rheumatology Society (ERE-EPERE), with two cohorts and a total number of approximately 3.000 patients.^18–22^ From this database, that included mainly patients from tertiary referral centers, a high percentage of seronegative patients was confirmed (48%). Despite the relatively low percentage of seropositive patients, 41% of patients had erosive disease at evaluation while various comorbidities were also common.

As expected, considering the inclusion of a large number of patients from hospital centers, among 2491 RA patients included, 42% patients were receiving biologic DMARDs at the time of evaluation (referral bias). 82% of patients were receiving csDMARDs (most commonly methotrexate at a median dose of 12.5 mg/week) either as monotherapy or as combination csDMARD (11%) therapy. One could argue that median MTX dose is “suboptimal”, and this could be discussed within Greek rheumatology community in order to be improved in coming years. Corticosteroids were used in 40% of patients at a median dose of 5 mg/day. In this cohort, an almost equal use on TNFα inhibitors and non-TNFα inhibitors was found. Biologic monotherapy was used in one out of four patients (26%) on bDMARDs, mainly due to previous side effects of csDMARDs or more rarely due to multiple co-morbidities prohibiting the use of csDMARDs.

During the initial cross-sectional phase of the study, one third of the RA patient population had achieved DAS28-ESR defined remission (34%) while an additional 18% was in low disease activity. It should be noted that the mean DAS28-ESR activity score had decreased from 4.9 at diagnosis to 3.1 at the time of evaluation, indicative of a major response.

## CO-MORBIDITIES

A major step towards improvement in the care of systemic inflammatory diseases was the recognition of co-morbidities as a contributor in long term outcomes. Available data for the current burden of comorbidities in Greek RA patients comes from several cohort studies (controlled or not), mainly for cardiovascular diseases^23–40^, the HeRBT and the RA-WG studies. Rates of first serious infection were 1.7–3.5 per 100 patient-years in TNFα-inhibitor therapies, with increasing age and high-dose glucocorticoids (>35 mg/week) being a major risk factors for serious infections in the Hellenic Registry of Biologic Therapies.^7^ Similar rates of serious infections (2.3/100 patient-years) were reported in the the RA-WG cohort (Thomas K et al., unpublished data). The rate of immunizations against influenza and pneumococcus was suboptimal with only one third of patients been vaccinated for both the year prior to the evaluation. Herpes zoster (HZ) is an increasingly recognized infection in RA patients with 6.7% of them reporting a past attack.

The prevalence of various cardiovascular risk factors was indirectly assessed from the electronic prescription database of biologic-treated patients.^17^ It was found that antihypertensive drugs were prescribed to 42% of patients, lipid-lowering drugs to 26% and antidiabetics to 12%. Similar results were reported from the RA-WG cohort.

## UNMET NEEDS

Achieving stringent targets correlates with better long-term outcomes in function, quality of life and work productivity, and consequently for optimization of the benefit provided by all therapeutic options. In a recent analysis form the HeRBT, 56% of RA patients on bDMARDs although improve their disease status still have sustained moderate disease activity (according to DAS28) in spite of bDMARDs switches. Most importantly, this status was associated with significantly worse 5-year outcomes (in terms of function and severe infections) compared to patients with sustained remission or low disease activity.^9^ Failure to effectively abrogate inflammation may have an adverse impact on the systemic consequences of inflammation such as in atherosclerosis and cardiovascular disease, and this should be further studied. Introduction of novel therapies (like the JAK inhibitors) in clinical practice will certainly help cover several of these unmet needs.

## CONCLUDING REMARKS AND PERSPECTIVE

Twenty years after the introduction of biologics in daily clinical practice, major advances in the improvement of the daily lives and outcome of patients with RA have been made creating a new therapeutic landscape for RA. Of note, efficacy data and drug survival rates reported for the bDMARDs-treated patients are for those patients (estimated to be 20–30% of the Hellenic clinical practice) with the most aggressive RA, mostly established and after multiple csDMARDs failure. This is a population that before the era of biologics had the worst prognosis with severe functional limitation and even shortened survival.^41^ It is in this group of aggressive RA that biologics have achieved better disease control, reduced orthopaedic surgeries and cardiovascular morbidity.^42,43^

Methotrexate remains the cornerstone of therapy RA and Greek rheumatologists were among the first in Europe to popularize its use. In spite of this, local and international experience suggests that the current target of therapy such as remission or low disease activity are achieved by only 40–50% of patients with early disease (less than 2 years), and only 20–30% of patients with longer duration. The introduction of biologics has increased these rates to 60–70% and 40–50% respectively. Among the patients with established disease on biologic therapy, 30–40% have moderate disease activity and 10–20% high disease activity despite the therapy.

In most cases, withdrawing biologic therapy in most patients on moderate disease activity further deteriorates their status, although this has not been studied formally. Importantly, data from the HeRBT assessing quality of life (QoL), suggest that in those patients with improvement in diseases activity after bDMARDs, HAQ and QoL improves significantly both in those achieving remission/low disease activity and in those who attain moderate disease activity.^5^ Better control of disease improves quality of life, productivity, decreases orthopaedic procedures and cardiovascular morbidity across a variety of countries both in Europe and in North America.^42,43^ The medical community and the patients cannot afford losing sight of the major impact that biologic therapy had on the lives of patients with RA.
